# Three-Dimensional Cross-Linking Network Coating for the Flame Retardant of Bio-Based Polyamide 56 Fabric by Weak Bonds

**DOI:** 10.3390/polym16081044

**Published:** 2024-04-10

**Authors:** Yunlong Cui, Yu Liu, Dongxu Gu, Hongyu Zhu, Meihui Wang, Mengjie Dong, Yafei Guo, Hongyu Sun, Jianyuan Hao, Xinmin Hao

**Affiliations:** 1 School of Materials and Energy, University of Electronic Science and Technology of China, Chengdu 610054, China; 202021030118@std.uestc.edu.cn (Y.C.); gdx706@163.com (D.G.); zhy172533297@163.com (H.Z.); jyhao@uestc.edu.cn (J.H.); 2 Systems Engineering Institute, Academy of Military Sciences, Chinese People’s Liberation Army, Beijing 100010, China; mhuiwang@126.com (M.W.); dmengjie2324@163.com (M.D.); guoyafei1010@163.com (Y.G.); 3 Binzhou Huafang Engineering Technology Research Institute, Binzhou 256617, China; shy@hfgf.cn

**Keywords:** flame retardant, synergistic effect, weak bond cross-linking, polyamide 56 fabric, polyelectrolyte complex

## Abstract

Weak bonds usually make macromolecules stronger; therefore, they are often used to enhance the mechanical strength of polymers. Not enough studies have been reported on the use of weak bonds in flame retardants. A water-soluble polyelectrolyte complex composed of polyethyleneimine (PEI), sodium tripolyphosphate (STPP) and melamine (MEL) was designed and utilized to treat bio-based polyamide 56 (PA56) by a simple three-step process. It was found that weak bonds cross-linked the three compounds to a 3D network structure with MEL on the surface of the coating under mild conditions. The thermal stability and flame retardancy of PA56 fabrics were improved by the controlled coating without losing their mechanical properties. After washing 50 times, PA56 still kept good flame retardancy. The cross-linking network structure of the flame retardant enhanced both the thermal stability and durability of the fabric. STPP acted as a catalyst for the breakage of the PA56 molecular chain, PEI facilitated the char formation and MEL released non-combustible gases. The synergistic effect of all compounds was exploited by using weak bonds. This simple method of developing structures with 3D cross-linking using weak bonds provides a new strategy for the preparation of low-cost and environmentally friendly flame retardants.

## 1. Introduction

Polyamide is a versatile material that has been applied in various industries, including textiles, electronics, construction and automobiles [1,2,3]. Its mechanical properties, abrasion resistance and electronic insulation make polyamide a popular choice in industry. Polyamide 56, also known as PA56, is a bio-based material obtained through a poly-condensation process of adipic acid and 1,5-pentane diamine. 1,5-pentane diamine is derived from starch fermentation. The unique odd–even asymmetric structure of PA56 results in many excellent properties, such as hygroscopicity and softness. However, its highly flammable nature with severe dripping limits the safety of PA56 [4,5].

There are three conventional strategies used to introduce flame retardants to fabrics for the improvement of flame retardancy, including physical blending, chemical bonding and surface treatment [6]. Although physical blending is the simplest method, the retardants readily migrate out when in use [7]. Chemical grafting of a flame retardant via different methods onto the fabric surface can improve thermal stability [8,9]. For example, covalent linkage of acrylamide (AM) with polyamide 66 (PA66) through chemical grafting [10], covalent grafting of phosphorus-containing acrylate on cellulose by argon plasma induction [11] or covalent modification of glycidyl methacrylate (GMA) with polyacrylonitrile (PAN) by ultraviolet induction [12] can ameliorate the limiting oxygen index (LOI) and peak heat release rate (pHRR) by altering the compact packing energy of local chains during crystallization [13]. Nevertheless, the chemical bonding method possibly changes the structure of macromolecules and damages the mechanical properties of fabrics.

Surface treatment by intermolecular weak bonding shows potential for making improvements in flame retardancy [14]. The self-assembly of flame retardants on the surface of fabrics can be driven by electrostatic interactions, hydrogen bonds and van der Waals forces [15,16,17]. This process results in the formation of various supramolecules, which enhance the thermal stability of the fabric [18,19]. Surface treatments applied to fabrics without specific softness requirements can also use metals or metal oxides to create a physical barrier [20].

Aza-heterocycles and their derivates are commonly used to modify fabric surfaces for flame retardancy through endothermic decomposition [21,22,23]. This process releases non-combustible gases, such as nitrogen oxides, NH_3_, CO_2_ and H_2_O, which dilute the concentration of oxygen and fuel gas on the fabrics [24]. They also encourage condensed phase char formation with nitrogen content. For example, melamine (MEL) has been used as a flame retardant with a typical 2D structure containing a high N content and is easily modified with other molecules to form new flame retardants [25]. Additionally, the molecules of MEL could interfere with the molecular motion during fabric melting to prevent dripping through intermolecular weak bonding [26].

Hyperbranched polyethyleneimine (hyPEI) is a positive polyelectrolyte containing many amino groups with the structure of one amino group linked to every two carbon atoms in three dimensions [27]. It can be used as a blowing agent and carbon source in ammonia-based flame retardants [28]. The large number of amino groups gives PEI a strong adsorption capacity [29,30,31], which can fix the flame retardant on the fabric surface through a large number of hydrogen bonds [32], and also enhances the flame retardant’s properties by promoting the absorption of carbon dioxide and moisture in the air [33,34], as well as facilitating the introduction of other flame-retardant components through chemical linkage or hydrogen bonding [35,36]. The hyperbranched structure of PEI enables the connection with other molecules to form the cross-linking network and enhance the flame retardant’s performance [37].

For enhanced flame retardancy, phosphates are often combined with ammonia-containing compounds inducing the formation of P-N cross-linking and the retention of P in the condensed phase to obtain a synergistic flame-retardant effect [38]. A common non-halogenated flame retardant is ammonium polyphosphate (APP), which is often complexed with nitrogen-containing compounds for a synergistic effect [39,40]. However, uneven dispersion of P-N bonding in the flame retardant is usually observed due to the limited water solubility, thus affecting the overall flame-retardant effect [41]. Sodium tripolyphosphate (STPP) is one kind of water-soluble linear polyphosphate with a low cost, low toxicity, environmental friendliness and good stability [42,43]. When STPP reacts with the oxygen in the air, the stable, non-toxic and harmless hydrated hydroxyapatite crystal particles can be generated to form the protective layer of fabrics.

We are, therein, assuming to combine hyPEI and MEL as the ammonia-based flame retardants and select STPP as the linker to connect hyPEI and MEL via P-N bonding or weak bonds in the polyelectrolyte flame retardants with a 3D supramolecular network. The hyperbranched structure of PEI constructs the basic framework of the 3D network. The good water solubility of STPP is expected to improve the dispersion of P-N bonding or weak bonds. A large number of reactive amino groups can provide sufficient opportunities for the connection with the fabric surface and the introduction of P elements to form a 3D network flame retardant through electrostatic interactions, hydrogen bonding and a phosphoramidation reaction, as well as by absorbing carbon dioxide and moisture from the air. Based on the very limited solubility of MEL and the simple three-step preparation process, some MEL molecules would be cross-linked with STPP and PEI in the 3D network and others would be packed on the surface of the 3D network by electrostatic interactions, hydrogen bonding, P-N bonding and π-π stacking so that MEL’s properties of cooling and gas blowing can be fully utilized.

## 2. Materials and Methods

### 2.1. Materials

PA56 fabric (61 g/m^2^) was supplied by Binzhou Huafang Engineering Technology Research Institute (China). Polyethyleneimine (PEI, MW = 10,000 g/mol, Aladdin, Shanghai, China), sodium tripolyphosphate (STPP, AR, Aladdin, Shanghai, China), melamine (MEL, AR, Aladdin, Shanghai, China), sodium citrate dihydrate (AR, Aladdin, Shanghai, China), citric acid monohydrate (AR, Aladdin, Shanghai, China) and sodium chloride (NaCl, AR, Aladdin, Shanghai, China), and acetic acid (AR, Yantai ShuangShuang Chemical Co., Ltd., Yantai, China) were used to prepare flame-retardant fabrics. All reagents were used directly after purchase without further purification.

### 2.2. Preparation of Polyelectrolyte Solution

The polyelectrolyte solution was prepared using PEI and STPP, with ratios set at 5 and 10 wt%, respectively. A total of 50 g of PEI was dissolved in 850 g of deionized water, utilizing magnetic stirring for an hour at room temperature, then adding 100 g STPP and stirring maintained for 1 h until a clarified homogeneous solution was obtained. Deionized water was prepared using a purifier (FST-I-20, Shanghai Fushite Environmental Protection Technology Co., Ltd., Shanghai, China). Other polyelectrolyte solutions with varying STPP contents of 2, 4, 6 and 8 wt% were also prepared using the same dissolution and mixing protocol.

### 2.3. Preparation of Melamine Solution

A series of aqueous MEL solutions were prepared under controlled pH and temperature conditions. Concentrations set were 0.5, 1, 2, 3 and 4 wt%. The solution of 4 wt% MEL was obtained by mixing 40 g of MEL and 19.2 g of acetic acid (1:1 molar ratio with MEL) into 940.8 mL of deionized water and heating to 60 °C with magnetic stirring for 1 h. MEL was dissolved by dropwise addition of acetic acid to pH 4.5. This protocol was similarly applied to prepare solutions with MEL concentrations set at 0.5, 1, 2 and 3 wt%, with the 0.5 and 1 wt% solutions stirred at room temperature, the 2 wt% solution prepared at 40 °C and the 3 wt% solution produced at 50 °C.

### 2.4. Preparation of Citric Acid Buffer

We prepared citric acid buffer solutions with pH values set at 3, 4, 5 and 6. These solutions were derived by incorporating defined concentrations of citric acid and sodium citrate into deionized water. Specifically, the pH 3 buffer was prepared by dissolving 16.8 g citric acid and 5.88 g sodium citrate in deionized water, yielding a final volume of 1 L. Subsequent to this, 11.69 g of NaCl (0.2 wt%) was added to increase the ionic strength. Buffers with other pH values were prepared using the same method.

### 2.5. Preparation of Flame-Retardant Fabrics

The fabric substrate was first immersed in the polyelectrolyte solution for 30 s, then passed through laboratory-scale padded rollers at a (65 ± 5)% wet pick-up rate and dried at 70 °C for 5 min. Subsequently, the polyelectrolyte-coated fabric was dipped in MEL solution for 30 s. After immersion in citrate buffer for 5 min, the fabric underwent rinsing with deionized water for the elimination of weakly adsorbed material. Finally, the coated fabric was subjected to drying at 70 °C for 20 min. We, here, named the fabric treated only with PEI, STPP and buffer as PA56@E/P (the coating was E/P), and the fabric treated with PEI, STPP, MEL and buffer as PA56@E/P/M-x, with the letter x representing the concentration of MEL (the coating was E/P/M-x).

### 2.6. Characterization

The structure of the flame retardant on the fabrics was obtained by attenuated total reflectance Fourier transform infrared (ATR-FTIR) spectrometer (INVENIO R, Bruker Optik GmbH, Ettlingen, Germany). Spectra were acquired with 16 scans per sample over a wavenumber range of 4000 cm^−1^ to 400 cm^−1^ at a resolution of 4 cm^−1^.

The surface morphology of the fabric fibers and combustion residues was examined using scanning electron microscopy (SEM, Apreo 2, Thermo Fisher Scientific, Waltham, MA, USA). Before imaging, the samples were sputter coated with a thin layer of gold. Micro-zone elemental analysis was performed using an energy dispersive X-ray spectroscopy (EDS) detector coupled to the SEM.

X-ray photoelectron spectroscopy (XPS) was performed using an X-ray photoelectron spectrometer (Escalab 250 xi, Thermo Fisher, Waltham, MA, USA) in a stainless steel vacuum chamber using an Al Kα radiation source.

Thermogravimetric analysis (TGA) was conducted using a Synchronized Thermal Analyzer (STA 449 F5, NETZSCH, Selb, Germany). Measurements were performed from 40 °C to 800 °C at a heating rate of 10 K/min under a N_2_ atmosphere, with a nitrogen gas flow rate of 20 mL/min.

Differential scanning calorimetry (DSC) was carried out on a Differential Scanning Calorimeter (DSC214, NETZSCH, Selb, Germany). Measurements were made using about 5 mg of the sample, which was heated from 0 °C to 300 °C under a N_2_ atmosphere, cooled down to 0 °C and then heated up again to 300 °C at a rate of 10 k/min.

Vertical flame testing was conducted using a vertical burner apparatus (M233M, SDL Atlas, Rock Hill, SC, USA) according to GB/T 5455-2014 standards [44]. Rectangular fabric samples of 300 mm × 100 mm were clamped in the sample holder and exposed to a methane pilot flame for 12 s. Each fabric formulation was tested in quintuplicate separately.

The LOI was determined using a limiting oxygen index analyzer (TTech-GBT2406-1, TES Tech Instrument Technologies, Suzhou, China) according to GB/T 5454-1997 standards [45]. Rectangular fabric specimens of 120 mm × 60 mm were clamped vertically in the test chamber and ignited from above. The LOI value corresponds to the minimal oxygen concentration for supporting sustained combustion.

Cone calorimetry was conducted using a cone calorimeter (FTT iCone mini, Fire Testing Technology Ltd., East Grinstead, UK) according to ISO 5660-1 standards [46]. Fabric specimens of 100 mm × 100 mm were stacked in quintuplicate to achieve the required sample thickness and tested at a heat flux of 35 kW/m^2^ with a 25 mm distance between the sample and the conical heating element.

Simultaneous thermogravimetry–infrared spectroscopy–mass spectrometry (TG-IR-MS) analysis was conducted using a Synchronized Thermal Analyzer (STA 449 F5, NETZSCH, Selb, Germany) coupled to a Fourier transform infrared spectrometer (VERTEX 70, Bruker Optics, Ettlingen, Germany) and mass spectrometer (QMS403C, Netzsch, Selb, Germany). Thermal decomposition measurements were performed from 40 °C to 800 °C at a heating rate of 10 K/min under N_2_ atmosphere. FTIR spectra were acquired from 4000 cm^−1^ to 400 cm^−1^ at a resolution of 4 cm^−1^ with 16 scans. The mass spectrometer operating parameters included an ion source temperature of 230 °C, ionization energy of 70 eV and m/z scan range from 1 to 300 amu.

Raman spectroscopic analysis of the combustion residue char was conducted using a laser Raman spectrometer (LabRAM HR Evolution, Horiba Scientific, Villeneuve d’Ascq, France) equipped with a 532 nm excitation laser. Spectra were acquired over the wavenumber range of 50–3400 cm^−1^ using a laser power of 1.5 mW focused to a 2 μm diameter spot size and 30 s integration time.

The tensile property of the fabrics was evaluated using an electronic tensile tester (YG026PC-250, Wenzhou Fangyuan Instrument, Wenzhou, China) following the guidelines of ASTM D5035 [47].

The bending length of the fabrics was measured using a fabric stiffness tester (TN11028-A, SafTonny, Dongguan, China) following the test methodology outlined in ASTM D1388 [48]. Durability testing was performed following the guidelines of GB/T 8629-2017 [49]. Fabrics were washed in an aqueous solution containing 2 g/L of commercial detergent at 40 °C using a medium-intensity washing protocol. After washing, the fabrics were hung to air dry.

## 3. Results and Discussion

### 3.1. Effect of Preparation Conditions on Fabric Weight Gain

**Figure 1:**
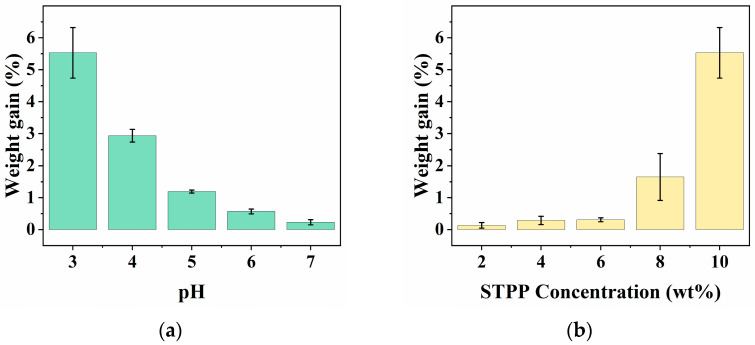
Effect of preparation conditions on weight gain, (**a**) pH, (**b**) sodium tripolyphosphate (STPP) concentration.

The protonation degree of the amine groups of PEI is dependent on pH [50], which consequently influences the electrostatic interaction between PEI and STPP and the deposition of the resultant polyelectrolyte flame retardant onto the fabric surface. The quantity of flame retardant on the fabric is usually positively correlated with the flame retardancy, and fabric weight gain is accordingly utilized as a characterization parameter of fabric coating and flame retardancy [51]. Fabric weight gain is the increased percentage of fabric weight after the surface treatment. Figure 1a shows the weight gain of fabrics after immersion in the solution containing 5 wt% of PEI and 10 wt% of STPP, and buffers at different pH values. The fabric weight gains increased as pH decreased. This result was ascribed to the increasing protonation of the PEI amino groups and phosphate groups of STPP with the increase in H^+^ concentration in the liquid environment, promoting the cross-linking of PEI with STPP and connecting the polyelectrolyte on the fabric surface through electrostatic forces and hydrogen bonds. The optimized pH value of the buffer was 3 and selected in our experiments.

The ratio of PEI and STPP also influences the cross-linking of raw materials and the surface coating of the flame retardant. We kept the amount of PEI and investigated the influence of STPP concentration on the fabric weight gain. Figure 1b shows that the weight gain of fabrics increased with the concentration of STPP. In our experiments, the maximum weight gain was obtained at the STPP concentration of 10 wt%, which was close to the saturated solubility of STPP.

### 3.2. Characterization of Coated Fabric

#### 3.2.1. FTIR

**Figure 2:**
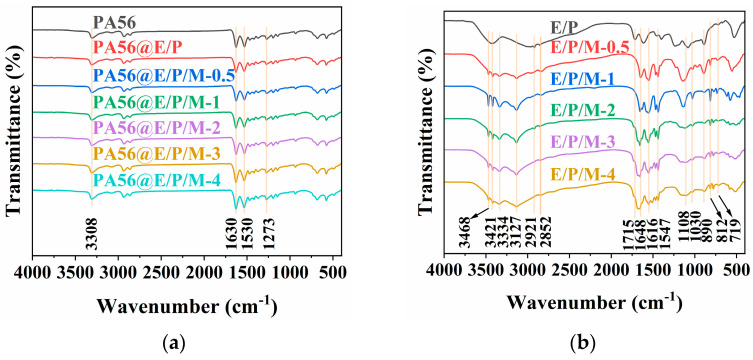
FTIR spectra, (**a**) fabrics, (**b**) surface coating. Fabrics treated with PEI, STPP and buffer only are named PA56@E/P (coated with E/P), and fabrics treated with PEI, STPP, MEL and buffer are named PA56@E/P/M-x, with the letter x representing the concentration of MEL (coated with E/P/M-x).

Attenuated Total Reflectance Infrared Spectroscopy (ATR-FTIR) can analyze samples in the surface and near-surface regions to study changes and trends in functional groups [52]. FTIR measures the changes in the fabric’s molecular structure after coating, and the results are displayed in Figure 2a. The peak at 3308 cm^−1^ corresponded to the N-H stretching vibration of the PA56 molecule, while the peaks at 1630, 1530 and 1273 cm^−1^ belonged to the amide I, II and III bands of the PA56 molecule, respectively [53]. No significant change was observed after coating, suggesting the coating in our experiments hardly changed the fabric molecules.

We examined the IR spectra of the surface coatings. The coatings were prepared on glass slides in the same way as for flame-retardant fabrics, as displayed in Figure 2b. The peaks at 3468, 3421, 3334 and 3127 cm^−1^ belonged to the N-H stretching vibrations of PEI and MEL. The C-H stretching vibrations of PEI were located at 2921 cm^−1^ and 2852 cm^−1^. The absorption peak at 1715 cm^−1^ corresponded to the C=O stretching vibration of residual citric acid in E/P. The N-H bending vibration of PEI at 1616 cm^−1^ was covered by 1648 cm^−1^ of MEL in E/P/M-x. The C-N bending and -NH_2_ stretching of MEL merged at 1547 cm^−1^. The peak at 1442 cm^−1^ was from a combination of -NCN stretching, -NH_2_ stretching, ring deformation and C-N stretching of MEL. The antisymmetric stretching vibrational absorption peak at 1187 cm^−1^ belonged to -PO_4_ in E/P, which red-shifted to 1140~1108 cm^−1^ in E/P/M-x due to hydrogen bonding. The absorption peak belonging to the MEL ring deformation was observed near 1030 cm^−1^ in E/P/M-x. The peaks at about 890 cm^−1^, 812 cm^−1^ and 719 cm^−1^ were ascribed to the antisymmetric stretching vibration of P-O-P, the toroidal profile variation of MEL and the telescopic vibrational peak of P-N, respectively. These results indicated the possibility of a connection between PEI and STPP.

#### 3.2.2. SEM and EDS

**Figure 3:**
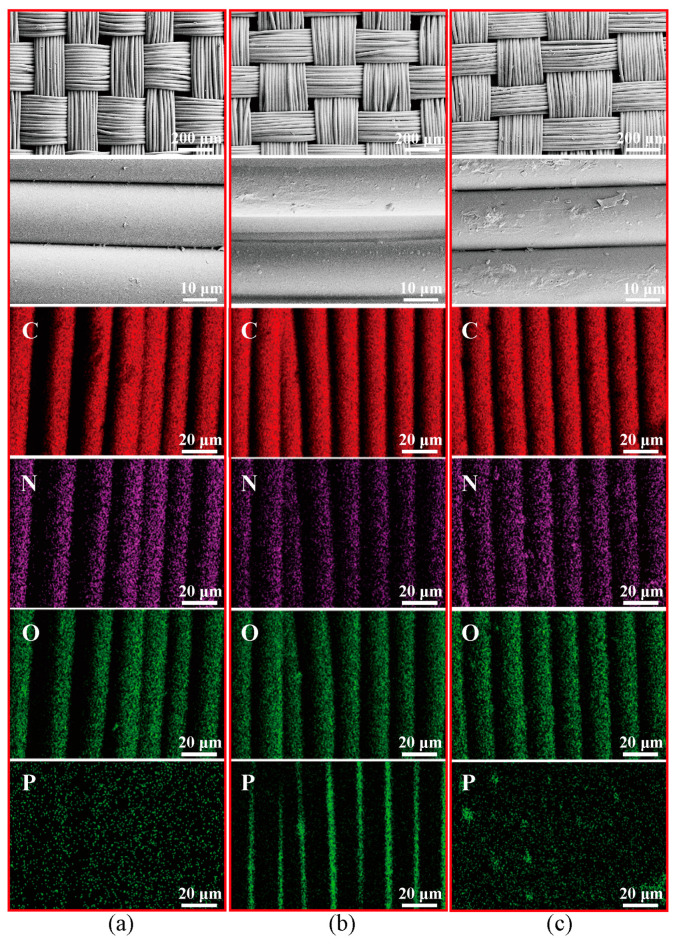
SEM and EDS (C, N, O, P elements) images of the fabric before and after coating, (**a**) PA56, (**b**) PA56@E/P and (**c**) PA56@E/P/M-4.

The surface morphology of the fabric was observed using SEM. A smooth surface with a clear and uniformly arranged network of individual fibers is observed for the original fabric in Figure 3a. PA56@E/P, as shown in Figure 3b, revealed a uniform coating that covered the fiber surfaces and filled the inter-fiber gaps, creating an adhesive bridging effect. These images indicate that PEI/STPP was successfully coated on the fabric. PA56@E/P/M-4, as shown in Figure 3c, depicted obvious flaky deposits, declaring the successful coating of the flame retardant.

**Scheme 1:**
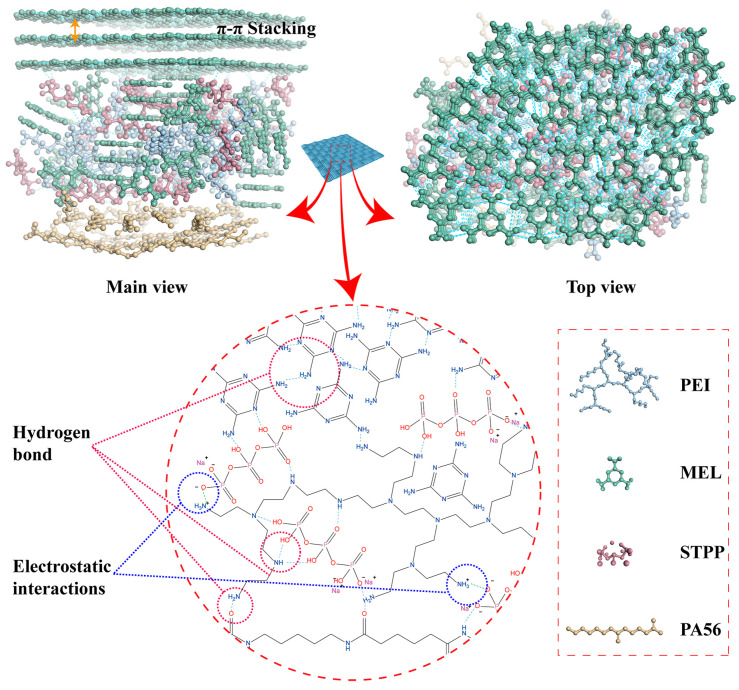
Illustration of flame retardant’s structure on the surface of PA56.

EDS elemental mapping of the fabric microstructure was also conducted. Figure 3 and Table 1 present the results. C, N and O signals showed a uniform distribution across the original PA56 fibers, as shown in Figure 3a. No signal of P was observed for original PA56. The PA56@E/P fabric, as shown in Figure 3b, revealed a distinct phosphorus signal localized on the fiber surface, confirming the coating of STPP. PA56@E/P contained 1.14% phosphorus. Figure 3c shows that the PA56@E/P/M-4 surface has the highest nitrogen content (18.09%) due to the presence of MEL, while no significant amount of elemental phosphorus is observed. It is possible that the lack of detectable phosphorus is due to its low content. It is important to note that EDS can only detect elements down to a micron-level depth; therefore, the elemental P content present on the surface is likely to be undetectable. This result demonstrated the structure of polyelectrolyte flame retardant conformed with our assuming as illustrated in Scheme 1. Some MEL molecules cross-linked with STPP and PEI in the bulk of polyelectrolyte flame retardant network and others were packed on the surface.

#### 3.2.3. XPS

**Figure 4:**
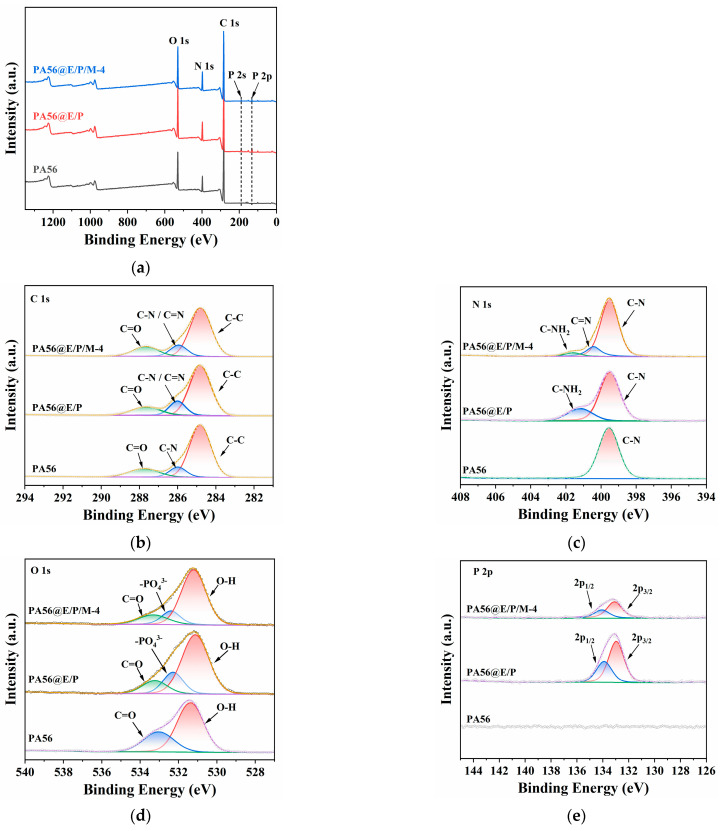
(**a**) Full XPS survey of PA56, PA56@E/P and PA56@E/P/M-4. High-resolution XPS spectra of C 1s (**b**), N 1s (**c**), O 1s (**d**) and P 2p (**e**).

X-ray photoelectron spectroscopy (XPS) measurements were performed on the PA56, PA56@E/P and PA56@E/P/M-4 fabrics. The results are shown in Figure 4. In Figure 4a, new peaks were observed at 132 eV and 189.6 eV, which belong to P 2p and P 1s, respectively. This indicates that the P element was successfully introduced into the flame retardant’s system. Figure 4b shows that the C element exists in the C 1s spectrum in the form of C=O, C-N/C=N and C-C. For the N 1s spectrum of Figure 4c, only one characteristic peak exists in PA56, which is attributed to C-N. In PA56@E/P, the peak located at 286 eV is attributed to C-NH2 of PEI. The N 1s spectrum of PA56@E/P/M-4 is divided into three parts, which are C-N (399.5 eV), C=N (400.4 eV) attributed to MEL and C-NH2 (401.7 eV). In the O 1s spectrum of Figure 4d, a characteristic peak attributed to the -PO4 group was found at 532.3 eV, which indicates the presence of STPP on the fabric surface. For Figure 4e, there are no characteristic peaks of elemental P on the PA56 surface, and the P 2p spectra of PA56@E/P and PA56@E/P/M-4 were divided into P 2p_3/2_ (133 eV) and P 2p_1/2_ (134 eV). XPS spectral analysis demonstrates the successful introduction of PEI, STPP and MEL. The P 2p spectra of PA56@E/P/M-4 in Figure 4e show the partial loss of STPP during the treatment of the fabric(PA56@E/P) with MEL solution. The XPS analysis reveals the presence of PEI, STPP and MEL, and their interactions result in incomplete removal from the fabric surface during rinsing.

### 3.3. Thermal Properties

**Figure 5:**
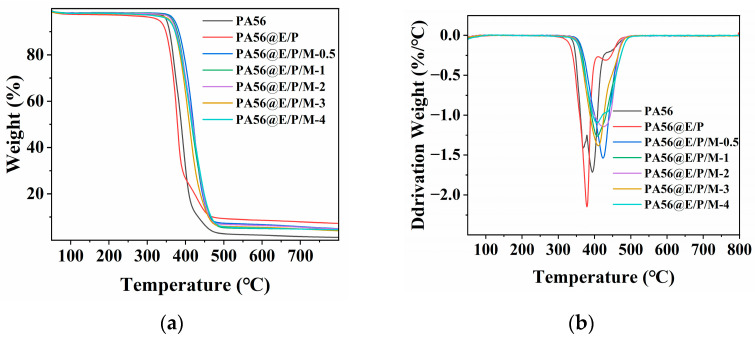
Thermogravimetric test results, (**a**) TG, (**b**) DTG.

The TG and DTG curves of the fabrics are displayed in Figure 5. The detailed results are summarized in Table 2. All fabrics exhibited a single-step degradation process between 350 °C and 500 °C. The T_5%_ (5% weight loss) values of PA56, PA56@E/P and PA56@E/P/M-4 were 350, 330 and 356 °C, respectively. The T_max_ (maximum weight loss) values of PA56, PA56@E/P and PA56@E/P/M-4 were 394, 378 and 405 °C, respectively. This result indicated that PEI/STPP accelerated the degradation of PA56 [18,28]. STPP decomposes at lower temperatures and catalyzes dehydration and carbonization [54]. The residue contents of PA56, PA56@E/P and PA56@E/P/M-4 were 1.2%, 7.3% and 4.5%, respectively. Both PEI/STPP and PEI/STPP/MEL can promote the char formation of PA56. Noteworthily, the T_5%_ and T_max_ values of PA56@E/P/M-x were visibly higher than those of both PA56 and PA56@E/P, and slightly decreased as the content of MEL increased. These findings suggested that the addition of MEL enhanced the thermostability of PA56 through cross-linking with other flame retardants and enough MEL can also promote the degradation of PA56 [55,56]. Additionally, the residue contents of PA56@E/P/M-x were less than those of PA56@E/P, possibly due to the generation of non-combustible gases.

**Figure 6:**
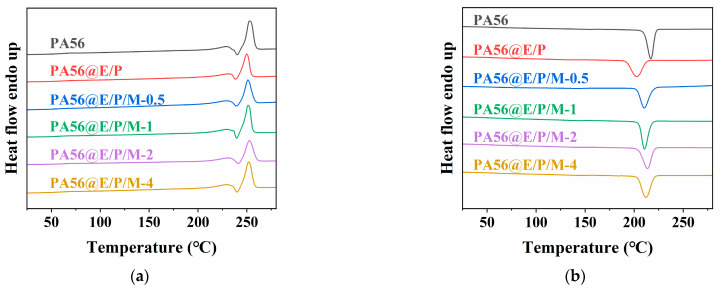
DSC curves under N_2_ atmosphere, (**a**) second heating curve, (**b**) cooling curve.

DSC curves of PA56, PA56@E/P and PA56@E/P/M-x are displayed in Figure 6, and the specific data are shown in Table 3. All fabrics showed a typical broad asymmetric melting peak, a melting–recrystallization peak and a small melting peak in the second heating curves [57,58]. The incorporation of flame retardants did not significantly alter the melting temperature (T_m_), but it lowered the crystallization temperature (T_c_). This result demonstrated that the addition of the flame retardants will not change the structure of PA56 molecules but can interfere with PA56 molecular motion; thereby, the melt dropping will be prevented.

### 3.4. Flame Retardants’ Properties

#### 3.4.1. Vertical Burning Test and Limiting Oxygen Index (LOI)

Table 4 presents the results of the vertical burning test and LOI for the fabrics. No fabric kept burning after the removal of the fire. No melt dripping was observed for any modified fabrics except PA56@E/P/M-0.5.

PA56@E/P showed a damage length of 30 cm. The sample was entirely burned, suggesting PEI/STPP accelerated the degradation of PA56 compared with the pure PA56 fabric. With the addition of MEL, the damage length was gradually decreased, implying PEI/STPP/MEL enhanced the thermostability of PA56 so that the combustion and melt dropping were prevented.

The LOI values of PA56, PA56@E/P and PA56@E/P/M-4 were 20.9%, 21.7% and 23.2%, respectively, demonstrating the flame retardancy of PEI/STPP and PEI/STPP/MEL. Decreasing the content of MEL reduced the LOI values. This result indicated that a sufficient amount of MEL can cross-link better with other flame retardants to form an efficient 3D network flame-retardant layer.

#### 3.4.2. Cone Calorimetry Test

**Figure 7:**
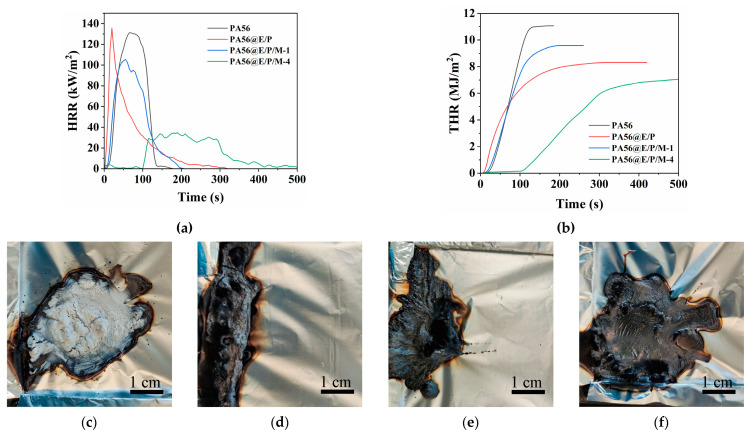
Cone calorimetry test curves, (**a**) heat release rate (HRR), (**b**) total heat release rate (THR). Residual image of fabric after cone calorimetry test, (**c**) PA56, (**d**) PA56@E/P, (**e**) PA56@E/P/M-1 and (**f**) PA56@E/P/M-4.

The cone calorimetry test simulated the realistic fire combustion behavior, further investigating the flame retardancy. Figure 7a,b display the heat release rate (HRR) and total heat release rate (THR) curves of the samples, respectively. Table 5 summarizes the results. In Figure 7a,b, pure PA56 exhibited an approximately linear increase in HRR and had the highest THR. Compared to pure PA56, the HRR curve of PA56@E/P became sharper and shifted towards shorter times. The HRR curve of PA56@E/P/M-x significantly shifted down and that of PA56@E/P/M-4 was the lowest and was delayed by about 110 s. The peak HRR (pHRR) values of PA56, PA56@E/P, PA56@E/P/M-1 and PA56@E/P/M-4 were 132.5 kW/m^2^, 139.9 kW/m^2^, 105.6 kW/m^2^ and 35.6 kW/m^2^, respectively, while the times to ignition (TTIs) of PA56, PA56@E/P, PA56@E/P/M-1 and PA56@E/P/M-4 were 22, 15, 16 and 113 s, respectively. The HRR values of PA56, PA56@E/P, PA56@E/P/M-1 and PA56@E/P/M-4 were 97.4 kW/m^2^, 27.2 kW/m^2^, 70.8 kW/m^2^ and 17.2 kW/m^2^, respectively. The slopes of the THR curves for PA56@E/P/M-1, PA56@E/P and PA56@E/P/M-4 reduced evidently. In particular, the THR curve of PA56@E/P/M-4 began to climb up after about a 110 s duration and with smaller total heat releases.

From the residue images of fabrics in Figure 7c–f, the carbonization of the treated fabrics was more prominent than that of pure PA56. The mass loss rate (MLR) values of all modified fabrics were lower than those of the unmodified fabrics. The MLR values of PA56@E/P, PA56@E/P/M-1 and PA56@E/P/M-4 were 1.7, 3.2 and 1.1 g/(s·m^2^), and the residues of PA56@E/P and PA56@E/P/M-4 increased visibly. The flame-retardant effect of PA56@E/P/M-4 was conspicuous among our experiments.

When analyzing the total smoke production (TSP), average CO yield (COy) and average CO_2_ yield (CO_2_y), it was found that the inclusion of flame retardants elevated smoke and incombustible gas release, while the introduction of MEL reduced the TSP, COy, and CO_2_y. This decrease correlated with the increasing of MEL’s content.

These results suggested that PEI and STPP promoted the decomposition of PA56, and the addition of a minor amount of MEL cross-linked incompletely with STPP so that the flame-retardant effect of STPP was reduced. With the increase in the amount of MEL, the addition of MEL can significantly delay the thermal decomposition time and reduce the heat release rate to a lower level. The very low HRR and pHRR values and long TTI of PA56@E/P/M-4 demonstrated that a sufficient addition of MEL can effectively cross-link with other retardants via weak interaction forces and covalent bonds to form the 3D network structure of the flame-retardant layer with MEL’s surface. This 3D network structure enhanced the thermostability and slowed down the combustion of fabrics. The carbonization of PEI/STPP can produce a carbonization layer to protect fabrics, and the decomposition of surface MEL can simultaneously generate non-combustible gases to lower the temperature and retard the combustion.

### 3.5. Flame Retardants’ Mechanical Properties

#### 3.5.1. TG-IR-MS Analysis

**Figure 8:**
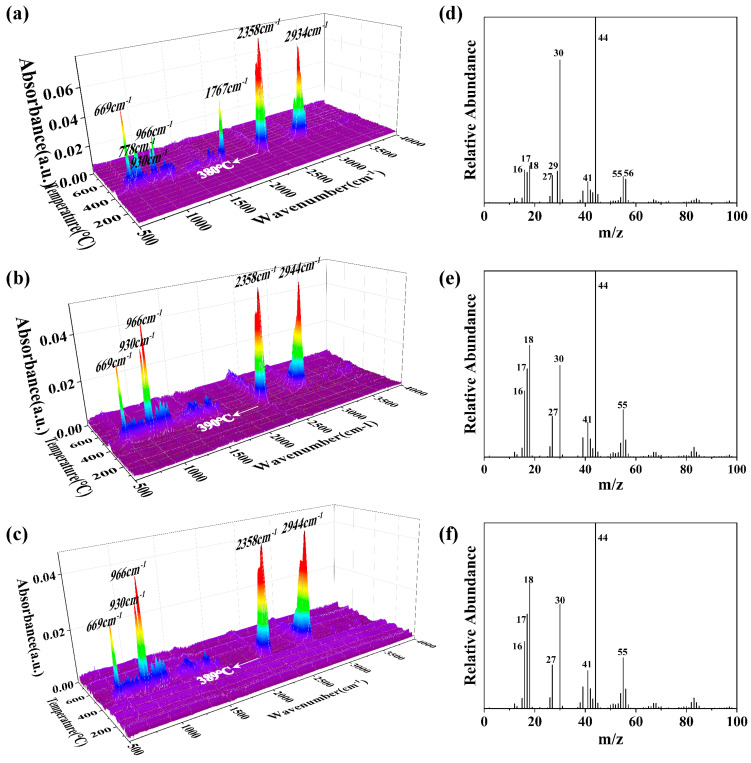
TG-IR and TG-MS spectra of the samples, (**a**) IR spectrum of PA56, (**b**) IR spectrum of PA56@E/P/M-1, (**c**) IR spectrum of PA56@E/P/M-4, (**d**) MS spectrum of PA56, (**e**) MS spectrum of PA56@E/P/M-1 and (**f**) MS spectrum of PA56@E/P/M-4. Where in (**d**–**f**), the major products such as m/z = 17, 18, 30, 44 correspond to NH_3_, H_2_O, C_2_H_6_^+^/CH_2_NH_2_^+^, and CO_2_, respectively.

TG-IR-MS analyses were conducted to measure the main pyrolysis gas products as shown in Figure 8. Distinct absorption peaks were observed at 669 and 2358 cm^−1^ for CO_2_ [59], and at 930 and 966 cm^−1^ for NH_3_ [60]. The absorption vibration peak, observed at 778 cm^−1^, corresponded to the out-of-plane deformation vibration of N-H. The peak at 1767 cm^−1^ was the stretching vibration of C=O, while the stretching vibration of alkane -CH_2_- was between 2934 cm^−1^ and 2944 cm^−1^ [61]. Thereby, the main products of unmodified fabrics after thermal decomposition included NH_3_, CO_2_, CH_2_O, small alkane molecules and compounds with amine groups. The pyrolysis products of PA56@E/P/M-1 and PA56@E/P/M-4 consisted of NH_3_, CO_2_ and small alkane molecules, with an increased proportion of NH_3_. The absorption peaks at 778 cm^−1^ and 1767 cm^−1^ were not significantly pronounced.

The MS spectra of fabrics are shown in Figure 8d–f, respectively. Signals at *m*/*z* = 16, 17 and 18 potentially corresponded to CH_4_, NH_3_ and H_2_O, respectively. The likely ionic signal at *m*/*z* = 27 could be generated by HCN^+^ or C_2_H_3_^+^. The signal at *m*/*z* = 29 was assigned to CHO^+^, while signals at *m*/*z* = 30 could originate from C_2_H_6_^+^ or CH_2_NH_2_^+^. The signal at *m*/*z* = 41 might represent C_3_H_5_^+^ or CH_3_CN^+^. CO_2_ was assigned to *m*/*z* = 44, and *m*/*z* = 55 and 56 could be signals from C_3_H_3_O and C_3_H_4_O^+^, which were possibly ionic fragments originating from cyclohexanone [62]. The less pronounced signals from *m*/*z* = 39, 42 and 43 might be assigned to C_3_H_3_^+^ (from aromatics), C_3_H_6_^+^ (olefins), C_3_H_7_^+^ (alkanes) or C_2_H_3_O^+^ (ketones). Signals from *m*/*z* = 82, 83 and 84 could potentially represent cyclohexene and its ring-opening products. Figure 8d reveals that the highest ion relative abundance corresponds to CO_2_, followed byC_2_H_6_^+^ / CH_2_NH_2_^+^. The TG spill gas of unmodified PA56 aligned with the TG-IR results. The other signals corresponding to CH_4_, H_2_O and NH_3_ were relatively weak. In Figure 8e,f, the increase in the *m*/*z* = 17 (NH_3_) ratio could be attributed to the escalated release of NH_3_ due to MEL’s addition. The diminished height of the *m*/*z* = 30 ion peak might be the effect of the interaction between CH_2_NH_2_^+^ and C_2_H_6_^+^ and the flame retardant. This result aligned with the absence of apparent absorption peaks at 778 cm^−1^ and 1767 cm^−1^ in Figure 7c and Figure 8b. The *m*/*z* = 18 (H_2_O) proportion in PA56@E/P/M-1 and PA56@E/P/M-4 escalated, ranking second to CO_2_. Therefore, the potential decomposition pathway of the PA56 fabric can be inferred as follows: the long PA56 molecular chain first breaks, generating cyclohexanone and cyclohexene, among others. It then undergoes further cleavage, ultimately producing small molecules such as CO_2_, H_2_O and NH_3_.

#### 3.5.2. Raman Analysis

**Figure 9:**
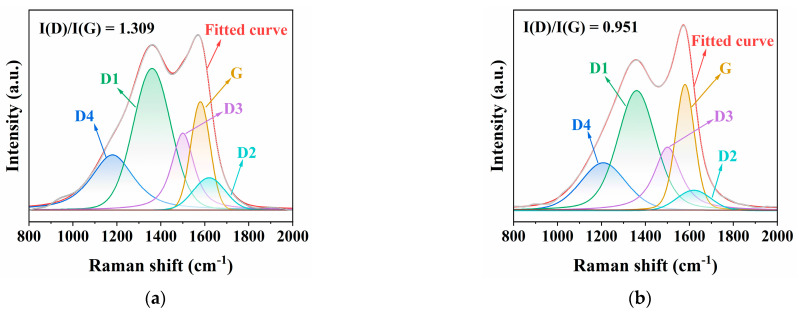
Raman spectra of combustion residual carbon, (**a**) PA56, (**b**) PA56@E/P/M-4. D1 (D) band: 1360 cm^−1^, G band: 1580 cm^−1^.

Raman spectroscopy is an effective method for analyzing the degree of graphitization of residual carbon. Previous studies have categorized the Raman spectra into the D band at 1360 cm^−1^ and the G band near 1580 cm^−1^. It is important to note that this method of peak fitting has limitations, as demonstrated in some studies. For this reason, in our study, we categorized the Raman spectrum from 800 cm^−1^ to 2000 cm^−1^ into D4, D (D1), D3, G and D2 bands [63], as shown in Figure 9. The I(D)/I(G) value was 1.309 in PA56 and reduced to 0.951 in PA56@E/P/M-4. The lower value of I(D)/I(G) suggested a higher degree of graphitization and a more stable carbon product. The stable carbon layer with a high degree of graphitization as a physical barrier on the fabric surface can effectively inhibit flame propagation and oxygen intrusion, thereby achieving effective flame retardancy.

#### 3.5.3. SEM of Residual Carbon

**Figure 10:**
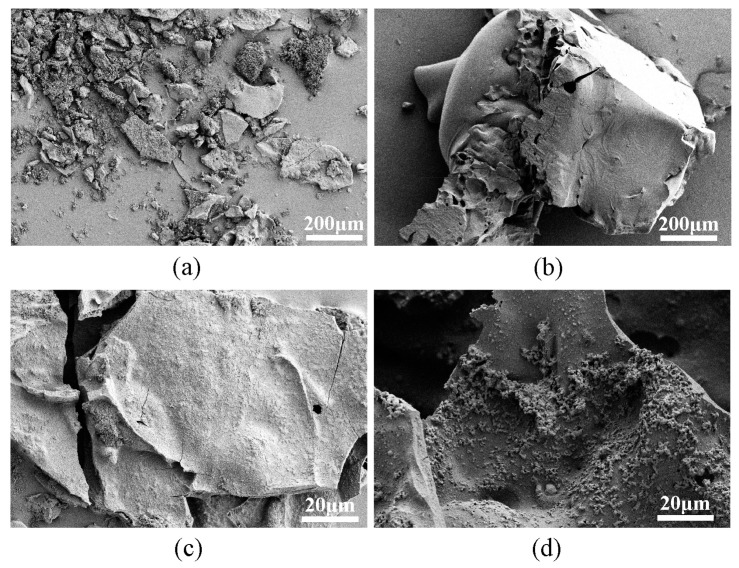
SEM images of combustion residual, (**a**) and (**c**) PA56, (**b**) and (**d**) PA56@E/P/M-4.

SEM analysis was also utilized to characterize the morphology of combustion residues. As shown in Figure 10a,c, the residual char of the untreated PA56 fabric appeared highly fragmented with smaller particle sizes lacking a continuous structure. The char surface exhibited a smooth topography without apparent porosity. The residues of PA56@E/P/M-4 exhibited the expanded and porous morphologies shown in Figure 10b,d resulting from the swelling effect of decomposition gases such as N_2_ and NH_3_ [64].

#### 3.5.4. FTIR of Char Residual

**Figure 11:**
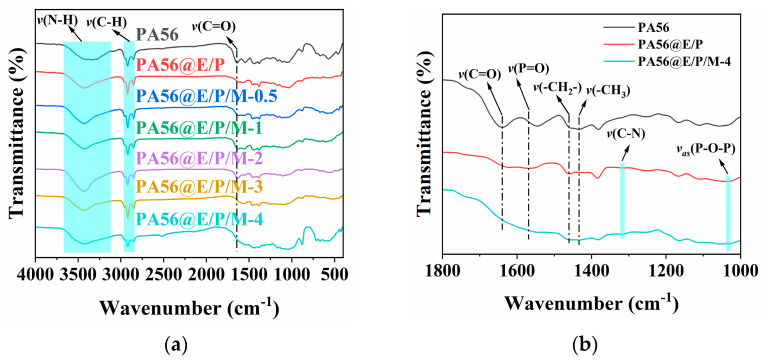
Infrared spectra of residual, (**a**) FTIR spectra of all samples, (**b**) FTIR spectra of PA56, PA56@E/P and PA56@E/P/M-4 from 1800 to 1000 cm^−1^.

The residual charcoal was also measured by FTIR and results are shown in Figure 11. No obvious difference was observed for PA56/E/P/M-x (x = 0.5~4), but the spectra of PA56, PA56@E/P and PA56@E/P/M-4 differed in some way. The peak near 1571 cm^−1^ (stretching vibration of P=O) appeared for PA56@E/P and PA56@E/P/M-4. The peaks near 1320 cm^−1^ (C-N stretching vibration of aromatic amine) and 1031 cm^−1^ (antisymmetric telescopic vibration of P-O-P) were found for the residues of PA56@E/P and PA56@E/P/M-4. No characteristic peaks associated with MEL were detected for the residue of PA56@E/P/M-4, suggesting the generation of the gas phase by the decomposition of flame retardants.

### 3.6. Mechanical Properties and Durability

The effect of flame retardant treatment on the fabric’s mechanical properties was assessed using breaking strength and bending length tests. The results are listed in Table 6. The mechanical properties of fabrics showed no prominent change before and after treatment by flame retardants, implying the flame retardant treatment in our experiment will not influence the mechanical properties of PA56.

Durability experiments were also performed and the results are shown in Table 7. We added 2 g/L of commercial detergent to water at 40 °C for medium washing, then hung the fabrics to dry. The flame retardant’s performance was then assessed using vertical burning tests. After washing 50 times, no melt dropping was observed, and char length increased very slightly, indicating the durability of PA56@E/P/M-4.

## 4. Discussion

The coatings were applied to the PA56 objects, as confirmed by the FTIR, SEM, EDS and XPS tests. The XPS tests successfully identified the C-NH_2_ of PEI, the -PO_4_ group of STPP and the C=N of MEL. The reduction in the P elemental content was also observed (the -PO_4_ integral area of PA56@E/P/M-4 is smaller than that of PA56@E/P), which corresponds to the weaker P-O bond absorption peaks detected by FTIR. The possibility of P element non-detection is consistent with the partial absence of P element content in the XPS results, as EDS detection depth can reach several micrometers.

The TG results indicate that STPP accelerates PA56 decomposition, and fabric thermal stability and flame retardancy can be improved by reducing STPP content and introducing MEL. The vertical combustion test demonstrated that the use of PEI and STPP effectively prevents molten droplets, while the inclusion of MEL enables self-extinguishing. Additionally, the CONE test showed that the inclusion of MEL delays the time to ignition (TTI) and significantly reduces heat release (HRR).

Finally, the TG-IR-MS test revealed that CO_2_ and hydrocarbons are the primary products of fabric decomposition. The use of flame retardants increases the yield of NH_3_ and H_2_O, which helps to stop the combustion process. The study of residual char showed that the application of flame retardants produces a porous residual char, which was confirmed by SEM testing. Additionally, the char has higher stability, as demonstrated by Raman testing. The FTIR test indicated the sublimation and decomposition of MEL.

## 5. Conclusions

We successfully coated PA56 fabrics with PEI, STPP and MEL using a straightforward three-step method. These three compounds were cross-linked through weak interaction forces, resulting in a 3D cross-linking network coating of flame retardants with MEL appearing on the surface. The application of the coating improved the thermal stability of the fabrics and resulted in a significant increase in the residual amount in the TG test. No melt dripping was observed in the vertical burning test. The HRR, pHRR and THR were decreased by maximums of 82.4%, 73.2% and 37.6%, respectively. More thermally stable residual char was generated after pyrolysis compared to the untreated PA56. The 3D cross-linking network coating altered the thermal degradation pathway of the fabric, and PEI and STPP enhanced char formation, while MEL generated NH_3_ and H_2_O gases that dilute oxygen and combustibles. Therefore, we propose a flame-retardant mechanism primarily governed by a gas phase flame-retardant process. The P compound (STPP) catalyzed the breakage of the PA56 molecular chain, while PEI acted as a charring agent, synergizing with STPP to enhance charring ability. The large amount of MEL on the surface of the coating produced non-combustible gaseous products and further boosted the fabric’s charring ability. This mechanism enhanced the gas phase flame retardancy. This report provided a strategy for coating bio-based PA56 with a 3D cross-linking network using weak bonds. The resulting material exhibited good mechanical properties and efficient flame retardancy in a controlled and simple manner.

## Figures and Tables

**Table 1:** Element content on the surface of fabrics.

Sample	C (wt%)	N (wt%)	O (wt%)	P (wt%)
PA56	64.58	16.41	19.01	0
PA56@E/P	63.77	15.18	19.91	1.14
PA56@E/P/M-4	61.95	18.09	19.96	0

**Table 2:** TG test results under N_2_ atmosphere.

Sample	T_5%_ (°C)	T_max_ (°C)	Char residue at 800 °C (wt%)
PA56	350	394	1.2
PA56@E/P	330	378	7.3
PA56@E/P/M-0.5	370	423	5.0
PA56@E/P/M-1	366	407	4.6
PA56@E/P/M-2	364	425	4.9
PA56@E/P/M-3	358	410	4.1
PA56@E/P/M-4	356	406	4.5

**Table 3:** DSC test results under N_2_ atmosphere.

Sample	T_m1_ (°C)	ΔH_m1_ (J/g)	T_m2_ (°C)	ΔH_m2_ (J/g)	T_c_ (°C)
PA56	229.2	12.2	253	61.8	217
PA56@E/P	230.3	10.2	250.2	44.7	202.6
PA56@E/P/M-0.5	229.2	8.8	251.1	50.3	210.7
PA56@E/P/M-1	229.9	8.5	251.2	52.7	210.7
PA56@E/P/M-2	230.9	8.2	252.2	48.8	213.7
PA56@E/P/M-4	229.3	7.1	252	55	211.8

**Table 4:** Vertical burning results and limiting oxygen index (LOI) values.

Sample	Weight Gain (%)	After-Flame Time (s)	After-Glow Time (s)	Dripping	Char Length (cm)	LOI (%)
PA56	-	0	0	Yes	16.3 ± 1.2	20.9
PA56@E/P	10.1 ± 0.2	0	16.2 ± 3.7	No	30	21.7
PA56@E/P/M-0.5	6.4 ± 0.1	0	18.3 ± 5.0	Yes	20.4 ± 1.7	19.6
PA56@E/P/M-1	5.9 ± 0.2	0	0	No	14.7 ± 1.4	20.9
PA56@E/P/M-2	6.0 ± 0.1	0	0	No	13.8 ± 1.0	21.6
PA56@E/P/M-3	5.5 ± 0.4	0	0	No	12.9 ± 1.4	22.1
PA56@E/P/M-4	6.2 ± 0.2	0	0	No	12.3 ± 1.3	23.2

**Table 5:** Cone calorimetry test data.

Sample	TTI (s)	HRR (kW/m^2^)	pHRR (kW/m^2^)	THR (MJ/m^2^)	TSP (m^2^/m^2^)	COy (kg/kg)	CO_2_y (kg/kg)	MLR (g/(s·m^2^))	Residue (%)
PA56	22	97.4	132.5	10.9	59.0	0.0214	1.85	4.1	3.8
PA56@E/P	15	27.2	139.9	7.7	150.1	0.0331	1.59	1.7	17.2
PA56@E/P/M-1	16	70.8	105.6	9	134	0.0322	1.70	3.2	7.9
PA56@E/P/M-4	113	17.2	35.6	6.8	142.6	0.0197	1.52	1.1	16.0

**Table 6:** Mechanical properties of the fabrics.

Sample	Tensile Strength (N)	Elongation at Break (%)	Bending Length (cm)
PA56	450.0 ± 11.4	85.0 ± 6.5	3.89 ± 0.29
PA56@E/P	456.2 ± 11.5	83.5 ± 3.2	4.64 ± 0.14
PA56@E/P/M-4	451.6 ± 13.7	85.7 ± 5.3	3.95 ± 0.25

**Table 7:** PA56@E/P/M-4 vertical combustion test results after washing.

Number of Washings	After-Flame Time (s)	After-Glow Time (s)	Dropping	Char Length (cm)
1	0	0	None	12.3 ± 1.3
5	0	0	None	12.9 ± 1.5
10	0	0	None	13.2 ± 0.9
20	0	0	None	14.1 ± 1.6
50	0	0	None	15.2 ± 1.7

## Data Availability

Data are contained within the article.
